# Inflammatory bowel disease mimicking granulomatosis with polyangiitis: a case report

**DOI:** 10.1186/s13256-016-1000-x

**Published:** 2016-08-02

**Authors:** Samantha C. Shapiro, Armen Khararjian, Rebecca L. Manno

**Affiliations:** 1 Department of Medicine, Johns Hopkins University School of Medicine, 600 N. Wolfe Street, Harvey 808, Baltimore, MD 21287 USA; 2 Department of Pathology, Johns Hopkins Medical Institutions, 600 N. Wolfe Street, Baltimore, MD 21287 USA; 3 Department of Rheumatology, Johns Hopkins University School of Medicine, 5501 Hopkins Bayview Circle, JHAAC Room 1B.13, Baltimore, MD 21224 USA

**Keywords:** Inflammatory bowel disease, Granulomatosis with polyangiitis, Cytoplasmic anti-neutrophil cytoplasmic antibody (c-ANCA)

## Abstract

**Background:**

We report a case in which the extraintestinal manifestations of inflammatory bowel disease preceded development of gastrointestinal symptoms by nearly 9 months in the context of an unusual autoantibody panel, mimicking granulomatosis with polyangiitis. This case highlights the intricacies and overlap of autoimmune diseases, and illustrates an interesting clinical phenotype: cytoplasmic anti-neutrophil cytoplasmic antibody positive inflammatory bowel disease with predominantly extraintestinal manifestations. Perinuclear anti-neutrophil cytoplasmic antibody positivity has been frequently reported in association with inflammatory bowel disease, but cytoplasmic anti-neutrophil cytoplasmic antibody positivity is uncommon.

**Case presentation:**

A 54-year-old African-American man presented to our internal medicine resident clinic at the Johns Hopkins Hospital with several months of systemic inflammatory features: anterior uveitis, auricular chondritis, monoarthritis, fever, and weight loss. He did not have a primary care physician due to lack of health insurance and had been seen in our emergency department several times over the past year. These features fit nicely with a diagnosis of granulomatosis with polyangiitis, especially given positive cytoplasmic anti-neutrophil cytoplasmic antibodies. However, 9 months into his clinical course he developed hematochezia with perirectal abscess and fistula. A colonoscopy with biopsy confirmed a diagnosis of inflammatory bowel disease.

**Conclusions:**

This case highlights the fact that extraintestinal manifestations may precede gastrointestinal symptoms of inflammatory bowel disease for months, which may delay diagnosis if not understood and recognized. It further highlights an interesting disease phenotype that has not been widely reported, but may deserve further study. Lastly, the case stresses the importance of the internist in identifying a unifying diagnosis in a slowly evolving clinical process with the assistance of subspecialists. In this respect, the case is of interest to general internists, as well as rheumatologists and gastroenterologists.

## Background

We present a case in which the extraintestinal manifestations of inflammatory bowel disease (IBD) preceded development of gastrointestinal (GI) symptoms by nearly 9 months in the context of an unusual autoantibody panel. IBD most commonly presents with GI symptoms. However, our patient presented with several extraintestinal manifestations of disease and positive cytoplasmic anti-neutrophil cytoplasmic antibody (c-ANCA), which mimicked granulomatosis with polyangiitis (GPA). An extensive literature review revealed no similarly published case reports at the time of article submission.

## Case presentation

A 54-year-old African-American man presented to our resident clinic at Johns Hopkins Hospital for discharge follow-up after admission for wrist pain and a suspected viral gastroenteritis. He did not have a primary care physician due to lack of health insurance and had been seen in our emergency department several times over the past year.

He had been well until 7 months earlier when he developed unilateral pain and redness of his right eye. An ophthalmologic evaluation in our emergency department revealed anterior uveitis, which resolved with topical steroids. Five months later he sought care for right ear pain. Computed tomography (CT) imaging revealed inflammation of his pinna, consistent with auricular chondritis. This resolved spontaneously without treatment. Two months later, he again returned to our emergency department with pain and redness of his right wrist. He was diagnosed with cellulitis and empirically treated with oral clindamycin for 7 days with minimal improvement. Just 3 days later, he was admitted for sudden onset of fever and diarrhea. A viral etiology was suspected given the quick resolution of his symptoms and management with conservative therapy. He was discharged home within 48 hours with follow-up in our internal medicine resident clinic.

At his out-patient visit a few days after discharge, he had persistent swelling of his right wrist that limited his ability to operate motor vehicles at his job as a valet. Further questioning revealed a 27 kg (60 pounds) unintentional weight loss over the past 6 months. He denied epistaxis, cough, hemoptysis, chest pain, dyspnea, recurrent ocular symptoms, rash, low back pain, abdominal pain, frequent stools, melena, or hematochezia. He had no significant past medical or surgical history. His family history was unremarkable. He did not smoke tobacco, drink alcohol, or use illicit drugs.

On examination, he was a well-developed well-nourished black man who appeared comfortable. He was alert and fully oriented. His vital signs were within normal limits. He had no rash, oral ulcers, or cutaneous nodules. There was no lymphadenopathy. Sclerae were not injected. A comprehensive musculoskeletal examination revealed mild synovitis of his right wrist without overlying erythema but limited range of motion due to pain. Cardiac, pulmonary, abdominal, and neurologic examinations were unremarkable.

A laboratory evaluation during admission revealed iron deficiency anemia with hemoglobin of 10.1 g/dL, and a white blood cell count of 12,100 cells/mm^3^ with a normal differential. His albumin was low at 2.9 g/dL, alkaline phosphatase was elevated at 182 U/L, and there was mild transaminitis. There was microscopic hematuria: red blood cells (RBC) 55/high-power field (hpf); he had normal creatinine and no proteinuria. His inflammatory markers were elevated with C-reactive protein 11.1 mg/dL and erythrocyte sedimentation rate 124 mm/hour. His fecal lactoferrin was positive. A chest radiograph showed a small ill-defined patchy infiltrate in the upper lobe of his right lung. An infectious workup included the following negative studies: bacterial stool and blood cultures, human immunodeficiency virus (HIV) viral load, cytomegalovirus (CMV) serum polymerase chain reaction (PCR), gonorrhea and chlamydia urine PCR, stool *Clostridium difficile* toxin, and stool ova and parasites. Anti-nuclear antibody, anti-mitochondrial antibody, anti-smooth muscle antibody, rheumatoid factor, and anti-cyclic citrullinated peptide were negative. His complements were normal. C-ANCA was positive at a titer of 1:40 with elevated proteinase 3 by enzyme-linked immunosorbent assay (ELISA; 102.6 units). Perinuclear anti-neutrophil cytoplasmic antibody (p-ANCA) and myeloperoxidase by ELISA were negative.

With infection effectively ruled out, his clinical picture seemed most consistent with GPA. He was seen in consult by rheumatology, and started on prednisone 60 mg daily with marked improvement in symptoms and laboratory abnormalities.

**Fig. 1:**
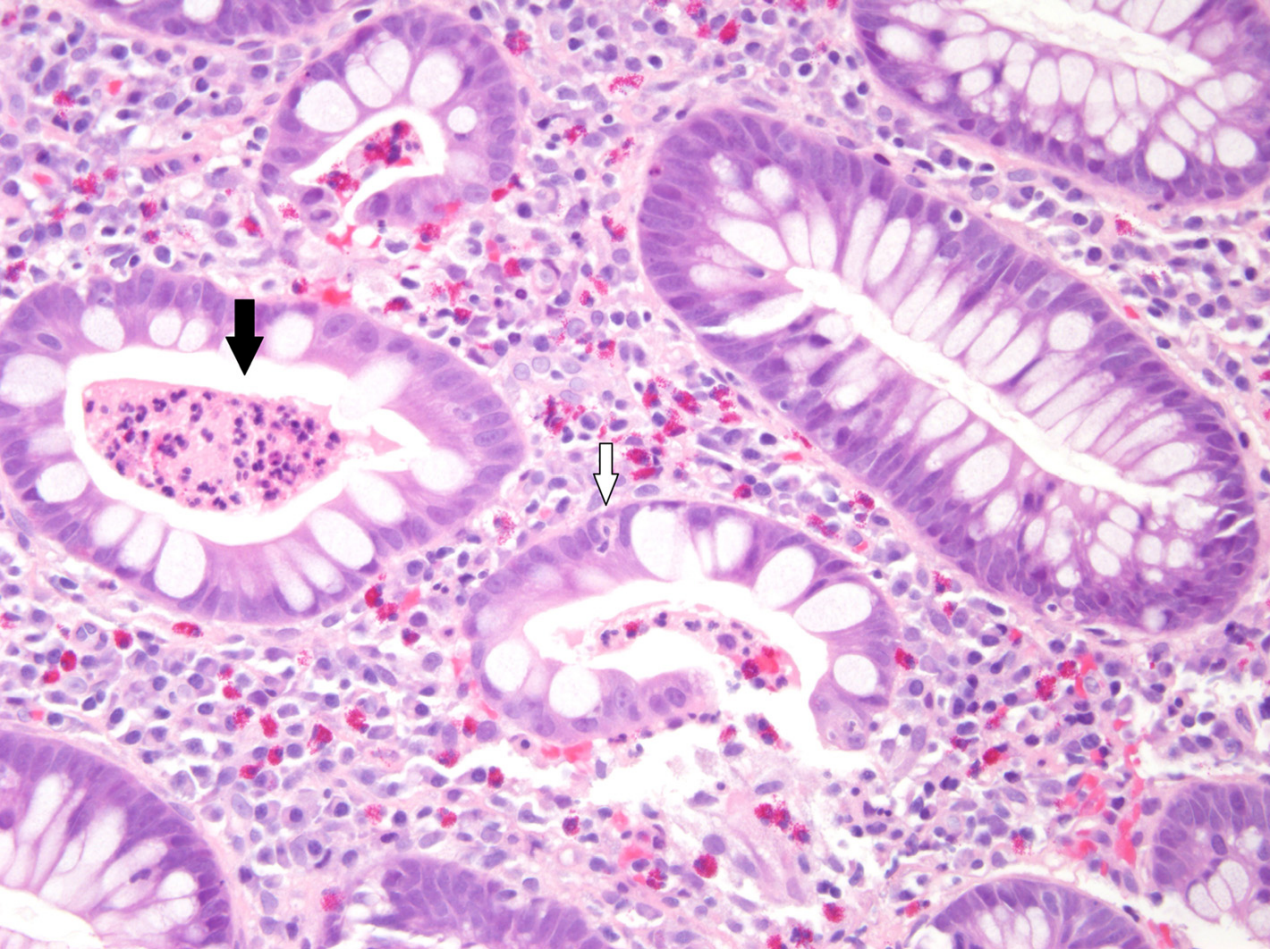
Descending colon biopsy. This histologic section from the descending colon, taken 9 months after initial presentation, shows a crypt abscess (*black arrow*) and cryptitis (*white arrow*). Enlarged at 20×

**Fig. 2:**
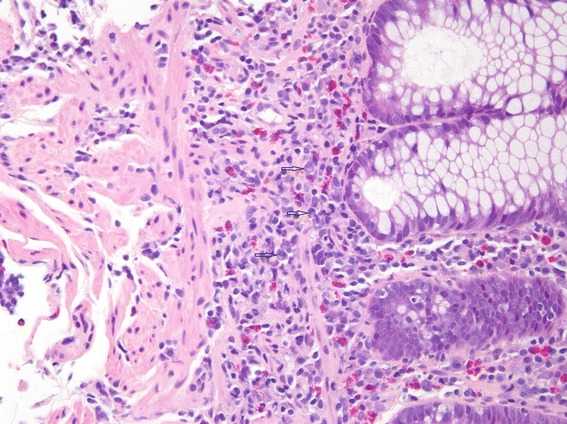
Rectum biopsy. This histologic section from the rectum, taken 9 months after initial presentation, shows basal plasmacytosis (*arrows*). Enlarged at 20×

However, 8 weeks later he developed hematochezia, left lower quadrant pain, and a perirectal abscess and fistula. A colonoscopy was performed and multiple biopsies were taken. Histologic examination of the biopsy from his descending colon (Fig. 1) showed cryptitis and crypt abscesses. A biopsy from his rectum (Fig. 2) showed early crypt distortion and basal plasmacytosis. In the absence of an infectious etiology, these findings were suggestive of a chronic colitis and/or IBD. There were no granulomas, vasculitis, or dysplasia.

Treatment for IBD was initiated with azathioprine and infliximab with healing of his fistula and continued clinical improvement. Therapy was well tolerated. For the past 1.5 years, he has been doing well on the same therapy with no further GI or extraintestinal manifestations of IBD.

## Conclusions

Our patient presented with a constellation of clinical and laboratory abnormalities over several months. Without health insurance, his complaints were evaluated piecemeal at sporadic emergency department visits. It was not until he established care with an internist and rheumatologist that the connection between these multisystem processes was revealed.

His clinical course was characterized by several striking inflammatory features: unilateral anterior uveitis, auricular chondritis, monoarthritis, fever, weight loss, microscopic hematuria, and c-ANCA positivity. These findings suggested a systemic autoimmune inflammatory etiology. The c-ANCA positivity further narrowed the differential to the two most likely diagnoses: GPA and IBD.

GPA is a small vessel vasculitis characterized by necrotizing granulomatous inflammation. It most commonly affects the upper and lower respiratory tract, kidneys, and lungs. Constitutional, ocular, otolaryngologic, musculoskeletal, GI, and neurologic symptoms may also be present [1]. Approximately 80 to 90 % of cases of GPA display ANCA positivity [1].

In our case, fever, weight loss, auricular chondritis, anterior uveitis, microscopic hematuria, monoarthritis, and c-ANCA positivity initially made GPA an attractive diagnosis. However, there were atypical features that did not fit well with this diagnosis. GPA is far more common in whites (~90 %) than in African-Americans with a prevalence of less than 4 % in non-white ethnicities [1]. Although uveitis has been rarely reported in GPA (0 to 10 % of patients), scleritis occurs far more frequently (16 to 38 % of patients) [2]. He had hematuria but lacked proteinuria, as is typical of renal involvement in GPA [3]. Lastly, our patient lacked obvious pulmonary features, although insurance limited evaluation with chest CT imaging. In one large series, pulmonary complaints were the primary reason for seeking treatment in 90 % of patients with GPA [3].

As his disease progressed and new features including abdominal pain and hematochezia emerged, his GI tract surfaced as the primary inflammatory target. Pathology from colonoscopy confirmed the diagnosis of IBD. This case is rare in that his presenting features were exclusively extraintestinal manifestations with c-ANCA positivity.

The reported frequency of extraintestinal manifestations of IBD ranges from 6 to 47 % [4]. A recent report from the Swiss Inflammatory Bowel Disease Cohort Study found that when present, extraintestinal manifestations preceded the diagnosis of IBD in only 25 % of cases (median time 5 months prior). The most common extraintestinal manifestations were peripheral arthritis (monoarthritis and oligoarthritis), aphthous stomatitis, axial arthropathy and ankylosing spondylitis, and uveitis [5]. Of note, the incidence of auricular chondritis was not reported in this study, nor has a strong association between auricular chondritis and IBD been widely reported in the literature. Limited case reports have noted auricular chondritis in patients who were ultimately diagnosed with both relapsing polychondritis and IBD [6–8].

ANCA positivity can be seen in several conditions that include but are not limited to vasculitides, other rheumatologic disorders, glomerular diseases, cystic fibrosis, colorectal cancer, autoimmune hepatitis, and a variety of infectious diseases. Of note, p-ANCA and atypical ANCAs are far more common in non-vasculitic processes then c-ANCA. ANCA positivity in IBD is not unusual as p-ANCA has long been associated with this diagnosis, but c-ANCA is far less common [9]. Some recent reports have associated c-ANCA with IBD and primary sclerosing cholangitis [10, 11]. In one study, c-ANCA via chemiluminescence assay was detected in 31 % of patients with ulcerative colitis (versus <2 % of Crohn’s disease), and was associated with shorter disease duration and more extensive colitis [10]. In another study, c-ANCA via chemiluminescence assay was also detected in 38.5 % of cases of primary sclerosing cholangitis compared to 10.6 % of liver disease controls, and this elevation was not exclusively related to underlying IBD [11].

GI symptoms are not uncommon in vasculitis. Of patients with systemic vasculitis, 10 % can present with ulcerations of the colon [12], and case reports have described GPA presenting with colitis [13, 14]. Furthermore, GI involvement is observed in approximately 20 to 30 % of patients with ANCA-associated vasculitis, but in one study vasculitis of the GI tract was evident on biopsy in only 30 % of patients with GI symptoms [12].

Given the overlap of clinical manifestations in this case, one must consider the possibility that the patient holds two separate diagnoses. A recent retrospective report from the French Vasculitis Study Group describes 11 such patients with coexisting ANCA-associated vasculitis and IBD. Seven patients with GPA also had Crohn’s disease (0.71 %), and four patients with eosinophilic GPA also had ulcerative colitis (0.78 %). In some cases, vasculitis and IBD were diagnosed simultaneously, but in others one diagnosis preceded the other, sometimes by as much as 10 years [15]. Longitudinal follow-up will reveal if our patient will evolve similarly.

In summary, this case represents an interesting disease phenotype: c-ANCA positive IBD with prominent extraintestinal manifestations preceding GI complaints. Failure to recognize this less common presentation of IBD may delay diagnosis. Furthermore, the significance of c-ANCA positivity in IBD is currently unclear, and further study of this entity may yield interesting information.

## Abbreviations

c-ANCA, cytoplasmic anti-neutrophil cytoplasmic antibody; CT, computed tomography; ELISA, enzyme-linked immunosorbent assay; GI, gastrointestinal; GPA, granulomatosis with polyangiitis; IBD, inflammatory bowel disease; p-ANCA, perinuclear anti-neutrophil cytoplasmic antibody; PCR, polymerase chain reaction
